# Intentional fatal metallic phosphide poisoning in a dog—a case report

**DOI:** 10.1186/s12917-015-0495-5

**Published:** 2015-07-23

**Authors:** Andras-Laszlo Nagy, Pompei Bolfa, Marian Mihaiu, Cornel Catoi, Adrian Oros, Marian Taulescu, Flaviu Tabaran

**Affiliations:** Department of Veterinary Toxicology, University of Agricultural Sciences and Veterinary Medicine, 3-5 Mănăştur Street, 400372 Cluj-Napoca, Romania; Department of Biomedical Sciences, Ross University School of Veterinary Medicine, Basseterre, St. Kitts, West Indies; Department of Animal Production and Food Safety, University of Agricultural Sciences and Veterinary Medicine, 3-5 Mănăştur Street, 400372 Cluj-Napoca, Romania; Department of Veterinary Pathology, University of Agricultural Sciences and Veterinary Medicine, 3-5 Mănăştur Street, 400372 Cluj-Napoca, Romania

**Keywords:** Canine, Phosphine, Poisoning, Veterinary forensic medicine, Lesions

## Abstract

**Background:**

Metallic phosphides are extremely toxic pesticides that are regulated in their usage. Information concerning the impact of metallic phosphides on human health is abundant. Data regarding the clinical pathology of phosphide poisoning in humans or domestic and wild animals is largely incomplete with only a few cases of metallic phosphide poisoning being reported every year, especially in humans. For the majority of cases reported in dogs the data are vague or incomplete. Here we report a complete and detailed description of pathological changes in a case of intentional metallic phosphide poisoning in a dog including an exhaustive examination of the brain.

**Case presentation:**

A 1 year old, male, Belgian Shepherd crossbreed dog with a clean medical history and no observed clinical signs prior to death, was submitted for post mortem examination. The dog was found dead by the owner. Near the body a suspect mix of bread, fat and a blackish powder was found. The owner announced the authorities and submitted the animal and the possible bait for forensic examination. At necropsy, multisystemic necrotic and degenerative lesions were observed. Histological exam confirmed the presence of necrotic and degenerative lesions of variable severity in all of the examined organs. The toxicological forensic examination revealed the presence of the phosphine gas in the gastric content and the bait.

**Conclusion:**

Metallic phosphide poisoning is a rarely reported entity, since the diagnosis of intentional poisoning with these compounds is a great challenge for forensic pathologists and toxicologists. To our knowledge, this is the first study describing the lesions completely in veterinary forensic toxicology. We assume that the toxic shows systemic endotheliotropism and damage of the endothelial cells responsible for the hemorrhagic lesions and for the secondary ischemic necrosis in various organs. This report will contribute to a better understanding of the pathogenesis in cases of acute metallic phosphide exposure in animals.

## Background

Metallic phosphides are effective rodenticides and insecticides widely used for over 100 years for the control of pests and to protect grain [1, 2]. Due to their toxicity to humans and domestic animals, the use of metallic phosphides (such as zinc and aluminum phosphides) is strictly regulated, being restricted to certified applicators. Following oral and inhalation exposures, metallic phosphides are classified as category I toxicants (high toxicity) in the United States of America [3] and acute toxicity category II (acute tox. 2) in the European Union [4]. In the past, before rigorous regulations were implemented, technical products were sold in shops, the baits for rodents were prepared directly by the consumers, a fact that led to accidental deaths in small children and pets. Moreover, numerous cases of suicide were reported [1].

Because of their extensive use as high efficient grain fumigant and rodenticide, low price and permissive legislation over the past two decades, a dramatic increase of human casualties following accidental or suicidal ingestion of phosphide pesticides was observed in developing countries as India and Iran [5–7]. Even if the information concerning metallic phosphides impact on human health is abundant, data regarding the incidence and clinical pathology of phosphide poisoning in domestic and wild animals is largely incomplete. This is mainly because the toxicosis is not well recognized by veterinarians, because the clinical signs and symptoms are nonspecific [8]. Moreover, reports of pathological changes in dogs poisoned with metallic phosphides are often vague and limited to gross descriptions or generally incomplete due to examination of only one or a few organs.

Here we describe the detailed post mortem, histopathological and toxicological findings from a dog that died following metallic phosphide poisoning.

## Case presentation

**Fig. 1:**
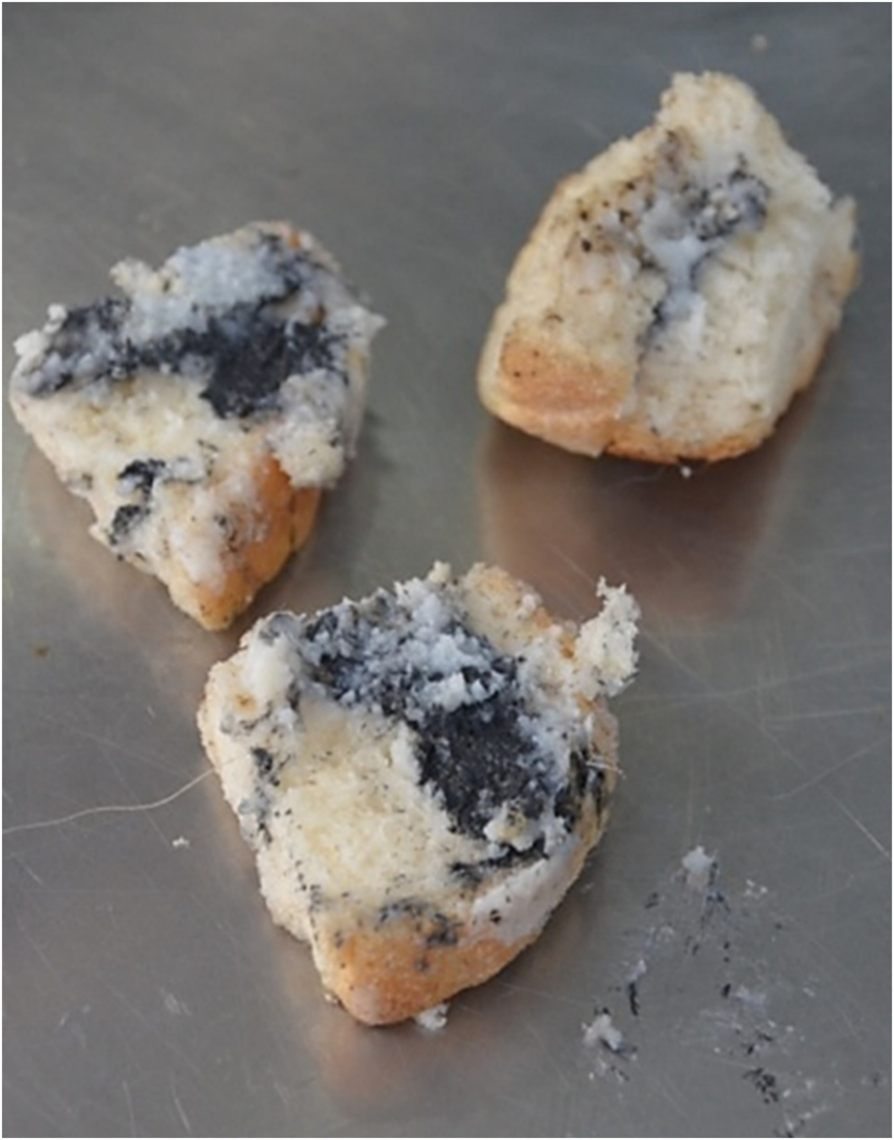
Gross aspect of the bait, a mix of bread, fat and a blackish powder

A 1-year-old male, Belgian Shepherd crossbreed dog with a clean medical history and no observed clinical signs prior to death, was submitted for post mortem examination, to the Pathology Department of the University of Agricultural Sciences and Veterinary Medicine Cluj-Napoca, Romania*.* The owner went to work in the morning, left the dog in the yard, and found him dead approximately 7 h later. Near the body there was a suspect mix of bread, fat and a blackish powder (Fig. 1). Since it was the third dog found dead over the last 5 years in the same yard, by the same owner, he announced the veterinary authorities and submitted the animal and the possible bait for forensic examination. Written informed consent was obtained from the owner of the dog for the publication of this report and any accompanying images.

A complete post mortem and histopathological examination were undertaken less than 24 h after death. For histology, the samples were fixed in 10 % buffered neutral formalin, embedded in paraffin, and 4micrometers sections were made. The slides stained with Hematoxylin–Eosin (H&E) stain. From the brain 5 coronal sections were made, starting from the frontal lobe to the cerebellum as previously recommended [9]. Thus, section 1 was through the telencephalon, section 2 interested the telencephalon and the anterior region of the diencephalon, section 3 and 4 were taken through the telencephalon and the posterior region of the diencephalon while section 5 was made through the metencephalon. The brain tissue slides were stained with H&E and Cresyl violet.

**Fig. 2:**
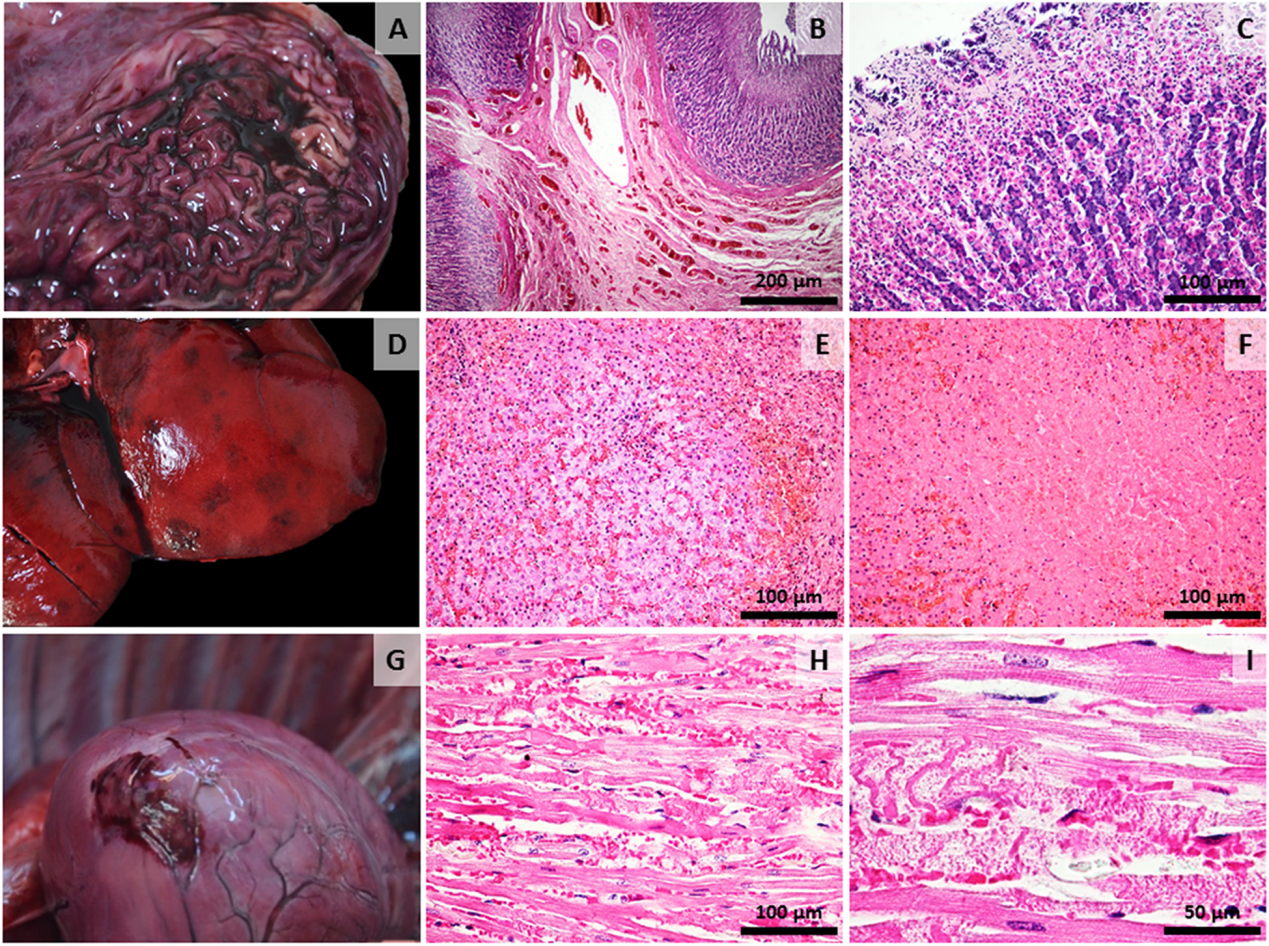
Gross and microscopic changes associated with acute metallic phosphide poisoning in the stomach, liver and heart. **a** Stomach, grossly there was diffuse congestion of the gastric mucosa, acute erosions in the fundic region and presence of digested blood covering the mucosa; (**b**) Stomach, diffuse congestion in the submucosa; most blood vessels are polled with red blood cells (H&E stain, original magnification 100X); (**c**) Stomach, superficial epithelial necrosis with desquamation; a few neutrophils and lymphocytes are visible adjacent to the eroded areas (H&E stain, original magnification 200X); (**d**) Liver, grossly the organ was congested, friable with multifocal areas of hemorrhage and necrosis; (**e**) Liver, microvesicular steatosis in the central area, diffusely congested sinusoid capillaries; in the center and in the upper left side of the image, clusters of inflammatory cells are visible (predominantly neutrophils); at the periphery (right side and upper left side of image) a mixture of coagulative necrosis and haemorrhage is observed (H&E stain, original magnification 200X); (**f**) Liver, central area of acute coagulative necrosis with loss of nuclei surrounded by congestion and haemorrhage (H&E stain, original magnification 200X); (**g**) Heart, apex, an area of myocardial necrosis and haemorrhage; (**h**) Heart, myocardial necrosis characterized by homogenization of sarcoplasm with loss of cross striations and micro haemorrhages(H&E stain, original magnification 200X); (**i**) Heart, fragmentation and loss of myocardial fibers, necrosis of the capillaries walls and haemorrhage (H&E stain, original magnification 400X)

Gross postmortem examination revealed multisystemic necrotic, haemorrhagic and degenerative lesions. In the proximal digestive tract (oral cavity, esophagus and stomach) a partially digested, haemorrhagic material was observed as a result of hematemesis. The stomach mucosa was severely congested, presenting in the fundic and pyloric regions multiple, acute erosions and approximately 200 ml of digested blood (Fig. 1a). The liver was enlarged, friable, and congested, presenting multifocal large foci of acute necrosis and haemorrhage (Fig. 1d). Kidneys were enlarged and congested. In the heart multifocal areas of myocardial necrosis and haemorrhages were seen, with one area visible close to the apex (Fig. 1g) and on the pericardium. In the brain, grossly meningeal congestion was the most striking feature (Fig. 2a). Multifocal petechial or ecchymotic haemorrhages were also observed in the parietal pleura and mediastinum. Severe bilateral pulmonary edema and petechial haemorrhages on the surface of the lungs were also observed (Fig. 2d), with presence of a frothy reddish fluid in the trachea (alveolar edema). Multifocal petechial haemorrhages were also visible in the cranial mediastinum, in the area of the thymus.

During the post mortem examination, samples of gastric content and urine were collected and submitted along with the presumptive bait for toxicological examination to the national reference laboratory of the National Sanitary Veterinary and Food Safety Agency of Romania in Cluj-Napoca. The silver nitrate test was used for the identification of the phosphine gas from the mix of bread, fat and a blackish powder as well as from the gastric content [10].

**Fig. 3:**
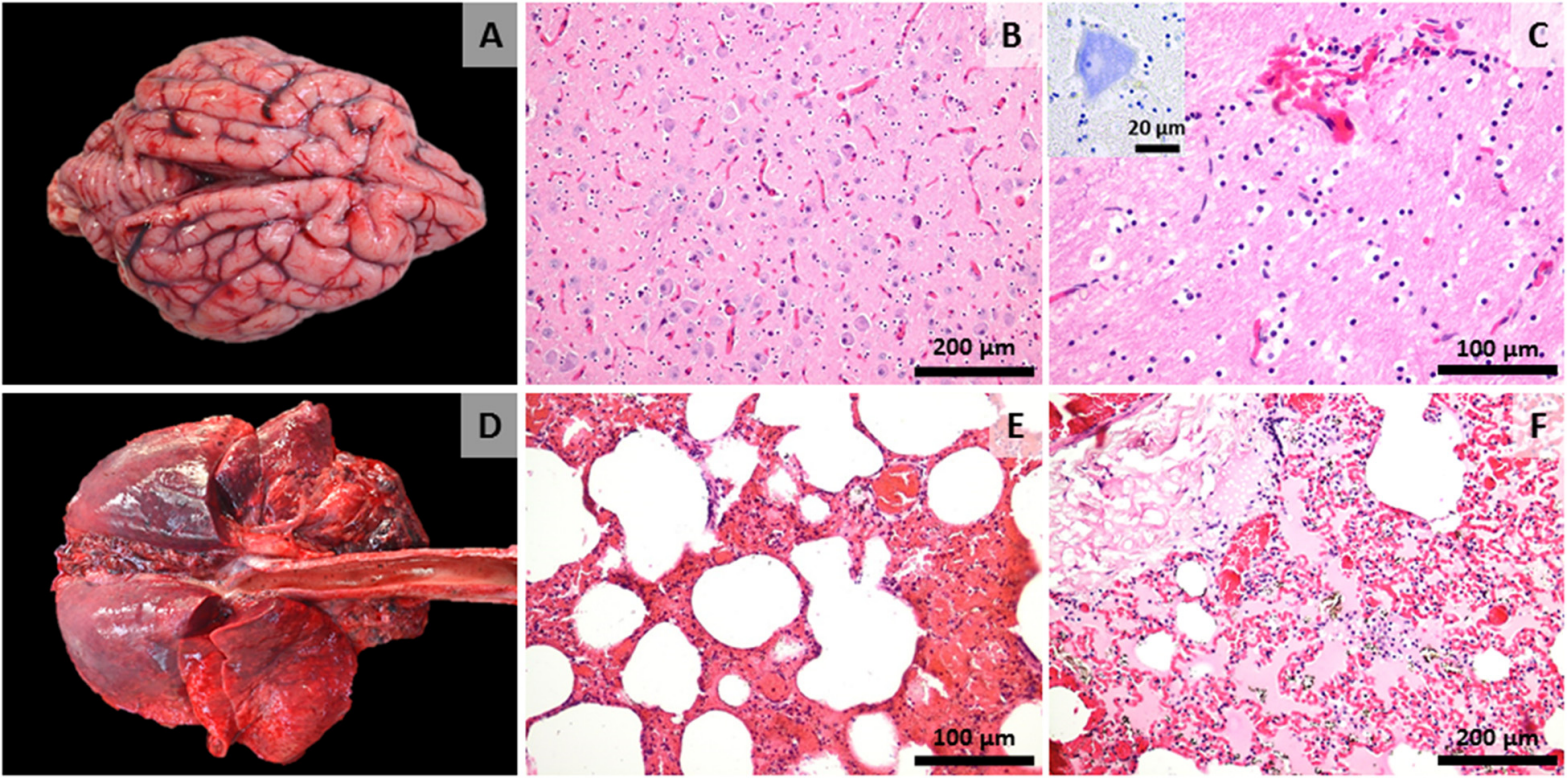
Gross and microscopic changes associated with acute metallic phosphide poisoning in the brain and lungs. **a** Brain, acute, diffuse meningeal congestion; (**b**) Brain, at the level of the telencephalon, small sized blood vessels are congested and there is mild gliosis (H&E stain, original magnification 100X); (**c**) Brain, an area of hemorrhage is observed (H&E stain, original magnification 200X), The inset shows a viable neuron with presence of Nissl substance (Cresyl violet stain, original magnification 1000X); (**d**) Lung, grossly, acute severe, diffuse pulmonary congestion, edema and multifocal petechial to ecchymotic hemorrhages are visible on the surface; (**e**) Lung, alveoli, congestion and hemorrhage with thickening of interalveolar septa (H&E stain, original magnification 200X); (**f**) Lung, severe, diffuse, alveolar congestion, edema and multifocal haemorrhage (H&E stain, original magnification 100X)

The predominant histopathologic finding in the stomach was severe congestion in the lamina propria and submucosa (Fig. 1b) and superficial epithelial necrosis with desquamation (Fig. 1c). In the liver, we observed: multifocal, acute coagulative necrosis with a few infiltrating neutrophils and macrophages (Fig. 1e) as well as multifocal areas of haemorrhage (Fig. 1f) and mild microvesicular mid-lobular steatosis. The renal cortex showed acute, diffuse tubular necrosis as the main histological finding, sometimes with loss or pyknotic nuclei or with discrete intracellular edema and mild vacuolar degeneration. The hearth showed multifocal areas of myocardial necrosis with loss of striations, fragmentation of myocardial fibers, degenerated nuclei and disrupted capillaries with consecutive haemorrhages (Fig. 2h and i). In the brain the most consistent change was congestion of blood vessels of all sizes (Fig. 2b). Multifocally the endothelium of small or medium sized blood vessels was discontinued resulting in discrete areas of haemorrhage, especially at the level of the telencephalon (Fig. 2c). Mild gliosis was observed multifocally throughout the different areas analysed (Fig. 2b) with no evidence of neuronal injury (Fig. 2c – inset). In the lung parenchyma histologically there was diffuse pulmonary congestion and edema, multifocal areas of capillary necrosis and consecutive haemorrhages (Fig. 3e and f). Overall, the histopathological lesions showed systemic endotheliotropism of the toxic evidenced by necrosis of blood vessels and consecutive haemorrhages as well as areas of ischemic necrosis of parenchymatous cells of different organs such as the liver, kidney or myocardium.

The toxicological examination confirmed the presence of the phosphine gas in the gastric content and in the bait. The urine was tested negative for the presence of phosphines.

The current report describes pathological and toxicological findings in an intentional poisoning with metallic phosphides in a dog. Poisoning with metallic phosphides in dogs can be accidental by direct ingestion of bait for different pests or by eating tissues of metallic phosphide-poisoned animals or it can be intentional. The forensic necropsy report has to be complete and clear and needs to contain all the information that may be relevant to a court case. The toxicity of these compounds is mainly due to the phosphine gases (PH_3_-known as phosphorus trihydride or hydrogen phosphide) liberated in contact with water and hydrochloric acid from the stomach [1]. Phosphine gas can be liberated even after the phosphides interact with the moisture in the air, or with the moist of the respiratory epithelium. Therefore acute poisoning with these compounds can occur after direct ingestion of metallic phosphides or indirect inhalation of the phosphine gas. Although in humans the phosphines are reported to produce toxicity following inhalation and oral exposure, currently only oral toxicosis was reported in dogs [8, 11].

Humans are very sensitive to phosphide compounds. This is exemplified by reports on the accidental occupational exposure in metallic phosphide manufacturing factories [2]. Because a part of the phosphine gas is also eliminated unchanged from the tissues or in the expired air, occupational exposure of physicians and veterinary staff members who treated or examined zinc phosphide poisoned patients has been reported [12, 13]. Owners should be informed about this risk of inhalation exposure to phosphine gas especially during transport of intoxicated animals to veterinary clinics. The transport should be done in well ventilated vehicles. There has been one reported case of a dog owner that required emergency medical care after his dog, after ingesting phosphide compounds, vomited in the car while the owner was driving to the veterinary clinic [8].

Most of the cases of zinc phosphide rodenticide toxicity reported in the veterinary literature resulted either in no clinical signs or in various clinical signs manifested in decreasing order as gastrointestinal signs, generalized malaise, central nervous system (CNS) signs, respiratory signs and cardiovascular signs [8]. These signs were in agreement with the gross and microscopical findings found by our study. The previously reported overall survival rate in cases of zinc phosphide rodenticide toxicity is 98.3 % (from 342 dogs) with only 5 dogs that didn’t survive: 1 was found dead by the owner, similar to our case, 2 dogs died during hospitalization, and 2 dogs were euthanized [8]. Currently, some type of decontaminant procedure remains the main treatment for phosphide toxicosis similar to most toxicoses in veterinary medicine [8]. The objectives of the decontamination procedures should include the reduction of gastric acidity, as this is having a substantial effect on phosphine gas production. Thus, as reviewed by [8], either administration of liquid antacids like magnesium or aluminum hydroxide and calcium carbonate or a solution of sodium bicarbonate should be performed before the gastric content is eliminated (either through lavage or emesis). Charcoal remains the option for decontamination.

The exact mechanism of action of the metallic phosphides is yet not known, but it was showed that PH_3_ inhibits cytochrome c oxidase which leads to mitochondrial dysfunction, a decrease of ATP (Adenosine triphosphate) production followed by metabolic shutdown and multiorgan dysfunction [5]. Metallic phosphides also increase the production of reactive oxygen species causing oxidative stress with all its pathologic effects on different cell components, at least in insects and rats [5]. As a result, depending on the dose, the lungs, liver, kidneys, heart, and CNS can be irreversibly affected [1, 5]. In our case, we believe that most of the lesions were caused by damage of endothelial cells in various organs (lung, liver, brain, myocardium and kidney). This resulted in hemorrhages and ischemic necrosis of parenchymal cells in the myocardium, liver kidney and heart.

## Conclusions

Metallic phosphide poisoning is reported sporadically in veterinary medicine. We describe here the gross and histopathological lesions associated with acute metallic phosphide exposure in a dog, showing that the toxic is mainly responsible for systemic acute necrotizing and hemorrhagic type of lesions, mainly targeting endothelial cells with secondary ischemic necrosis in the affected tissues.

## Abbreviations

H&E: Hematoxylin-Eosin
PH_3_: Phosphorus trihydride
CNS: Central nervous system
ATP: Adenosine triphosphate
